# Supplementary material for: Classification and Prognosis Analysis of Pancreatic Cancer Based on DNA Methylation Profile and Clinical Information
Source: Genes (Basel). 2022 Oct 21;13(10):1913. doi: 10.3390/genes13101913 (PMC9601656; doi:10.3390/genes13101913)
Supplement: Supplementary file 1 [file genes-13-01913-s001.zip › genes-1922944-Table S1.pdf]

Table S1. Example of partial methylation profiles after pretreatment

Gene.Symbol	TCGA.S4.A8RP.01	TCGA.IB.A6UG.01	TCGA.US.A776.01
A1BG	0.59065	0.5907	0.2609
ACSS3	0.1648	0.3682	0.2157
ADAM12	0.5187	0.4384	0.5310
ADAMTS18	0.4055	0.4141	0.3650
ADAP1	0.4286	0.5211	0.2703
ADCY1	0.2064	0.4376	0.1136
ADCY3	0.3366	0.4806	0.3305
ADH4	0.5180	0.7367	0.4173
ALPI	0.6066	0.5532	0.4839
ALX1	0.3484	0.3662	0.2939
ANKRD20B	0.4954	0.5062	0.5824
