# Supplementary material for: Classification and Prognosis Analysis of Pancreatic Cancer Based on DNA Methylation Profile and Clinical Information
Source: Genes (Basel). 2022 Oct 21;13(10):1913. doi: 10.3390/genes13101913 (PMC9601656; doi:10.3390/genes13101913)
Supplement: Supplementary file 1 [file genes-13-01913-s001.zip › genes-1922944-Table S2.pdf]

Table S2. Examples of top15 significant univariate cox regression results

Gene.name	Hazard.Ratio	95% Confidence interval	p.value
C11orf34	0.03	0-0.18	<0.001
IL20RB	0.03	0.01-0.14	<0.001
MYEOV	0.01	0-0.12	<0.001
ANKRD57	0.03	0-0.21	0.001
FAM83A	0.02	0-0.21	0.001
HIST1H2BK	0.02	0-0.22	0.001
PLAU	0.05	0.01-0.33	0.002
SLC39A2	0.03	0-0.29	0.003
CAPN2	0.01	0-0.21	0.004
EXTL1	0.01	0-0.22	0.004
FCRLA	0.03	0-0.33	0.004
HOXC12	36.64	2.94-457.35	0.005
CHST4	0.1	0.02-0.51	0.006
MAP4K5	0.14	0.04-0.56	0.006
