# Supplementary material for: Classification and Prognosis Analysis of Pancreatic Cancer Based on DNA Methylation Profile and Clinical Information
Source: Genes (Basel). 2022 Oct 21;13(10):1913. doi: 10.3390/genes13101913 (PMC9601656; doi:10.3390/genes13101913)
Supplement: Supplementary file 1 [file genes-13-01913-s001.zip › genes-1922944-Table S3.pdf]

Table S3. Top 15 significant results of multivariate cox regression analysis

Gene.name	Hazard.Ratio	p.value
IL20RB	0.024058797	<0.001
MYEOV	0.011066403	<0.001
ANKRD57	0.027997057	<0.001
FAM83A	0.028973563	0.001
HIST1H2BK	0.023579432	0.001
PLAU	0.052454276	0.001
C11orf34	0.040479933	0.002
EXTL1	0.008051021	0.003
SPATA21	0.052794781	0.003
SMG6	0.021086653	0.005
FCRLA	0.040093744	0.006
MAP4K5	0.148089138	0.006
SLC39A2	0.038290701	0.007
CAPN2	0.009838279	0.007
EVC	5.350443069	0.008

P-value corrected for multiple tests using the Benjamini–Hochberg method. The hazard ratio (HR) of the COX model shown. The HR is relative, and sites with value > 1 and < 1 are considered to be associated with poor and good prognosis, respectively.
