# Supplementary material for: Classification and Prognosis Analysis of Pancreatic Cancer Based on DNA Methylation Profile and Clinical Information
Source: Genes (Basel). 2022 Oct 21;13(10):1913. doi: 10.3390/genes13101913 (PMC9601656; doi:10.3390/genes13101913)
Supplement: Supplementary file 1 [file genes-13-01913-s001.zip › genes-1922944-Table S4.pdf]

Table S4. Top 20 differentially methylated genes among pancreatic cancer subtypes

Gene Name	DE-β	P	adjustP
MIR135B	-0.2454	5.15E-28	4.02E-26
CAPN8	-0.2683	4.45E-25	1.16E-23
FAM25A	-0.1329	4.27E-25	1.16E-23
CAPN2	-0.1055	3.46E-24	5.39E-23
HRH1	-0.1649	1.10E-22	1.44E-21
MTMR7	0.1595	1.72E-21	1.91E-20
SLAMF7	-0.1753	4.27E-20	4.17E-19
GSDMC	-0.2042	4.85E-20	4.21E-19
S100A10	-0.1218	8.34E-20	6.51E-19
ARNTL2	-0.1006	3.62E-19	2.57E-18
CCDC70	-0.1410	1.63E-16	9.79E-16
SNORD114.29	-0.1990	1.63E-16	9.79E-16
SLPI	-0.1375	1.90E-16	1.06E-15
MYEOV	-0.1251	5.30E-16	2.76E-15
HOXC12	0.1022	1.18E-15	5.13E-15
UCA1	-0.1927	1.18E-15	5.13E-15
KRTAP2.4	-0.2117	1.85E-15	7.61E-15
LOC728392	0.2163	3.42E-15	1.34E-14
SHROOM3	-0.1247	5.67E-15	2.11E-14
ISG20L2	-0.1166	6.08E-15	2.16E-14
