# Supplementary material for: Classification and Prognosis Analysis of Pancreatic Cancer Based on DNA Methylation Profile and Clinical Information
Source: Genes (Basel). 2022 Oct 21;13(10):1913. doi: 10.3390/genes13101913 (PMC9601656; doi:10.3390/genes13101913)
Supplement: Supplementary file 1 [file genes-13-01913-s001.zip › genes-1922944-Table S5.pdf]

Table S5. Characteristic pathways of methylated genes in subtypes

cluster	MyList	Biological Process (GO)
1	*LOC728392*	None
1	*EVC*	GO:0003416 endochondral bone growth;GO:0098868 bone growth;GO:0045880 positive regulation of smoothened signaling pathway
1	*HOXB4*	GO:0048103 somatic stem cell division;GO:0048539 bone marrow development;GO:0060218 hematopoietic stem cell differentiation
2	*CAPN8*	GO:0007586 digestion;GO:0006508 proteolysis;GO:0019538 protein metabolic process
2	*CSTA*	GO:0018149 peptide cross-linking;GO:0030216 keratinocyte differentiation;GO:0009913 epidermal cell differentiation
2	*GSDMC*	GO:0070269 pyroptosis;GO:0042742 defense response to bacterium;GO:0009617 response to bacterium
2	*HEBP1*	GO:0007623 circadian rhythm;GO:0048511 rhythmic process;GO:0008150 biological_process
2	*HRH1*	GO:0032962 positive regulation of inositol trisphosphate biosynthetic process;GO:0032960 regulation of inositol trisphosphate biosynthetic process;GO:0071420 cellular response to histamine
2	*KRTAP2.4*	None
2	*MAP4K5*	GO:0000165 MAPK cascade;GO:0006468 protein phosphorylation;GO:0016310 phosphorylation
2	*MIR135B*	GO:1904754 positive regulation of vascular associated smooth muscle cell migration;GO:1905564 positive regulation of vascular endothelial cell proliferation;GO:0014911 positive regulation of smooth muscle cell migration
2	*SLAMF7*	GO:0042267 natural killer cell mediated cytotoxicity;GO:0002228 natural killer cell mediated immunity;GO:0001909 leukocyte mediated cytotoxicity
2	*UCA1*	GO:0010468 regulation of gene expression;GO:0060255 regulation of macromolecule metabolic process;GO:0019222 regulation of metabolic process
2	*SNORD114.29*	None
