# Supplementary figures and images for: Classification and Prognosis Analysis of Pancreatic Cancer Based on DNA Methylation Profile and Clinical Information
Source: Genes (Basel). 2022 Oct 21;13(10):1913. doi: 10.3390/genes13101913 (PMC9601656; doi:10.3390/genes13101913)

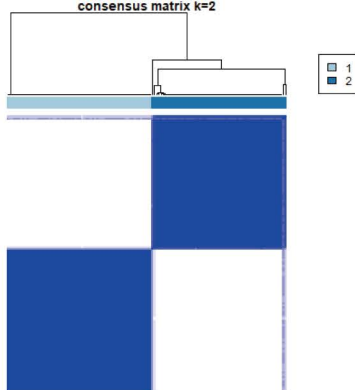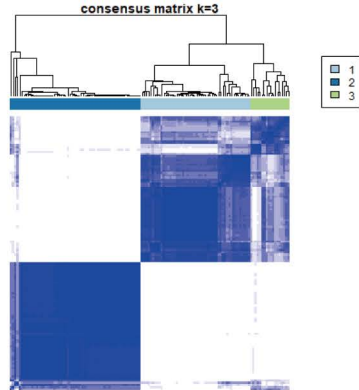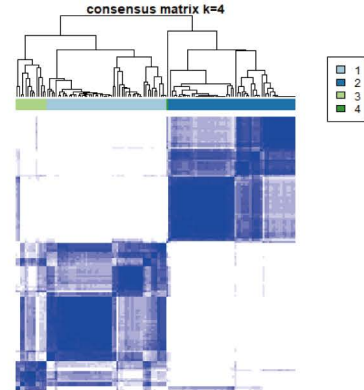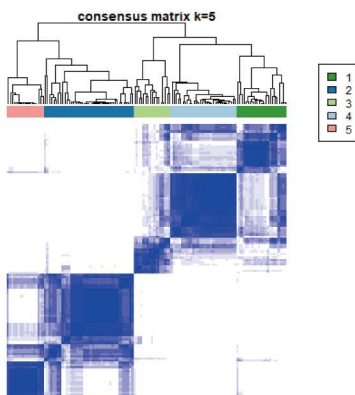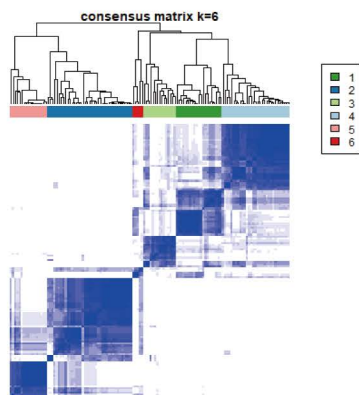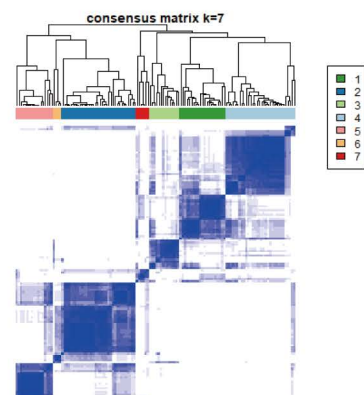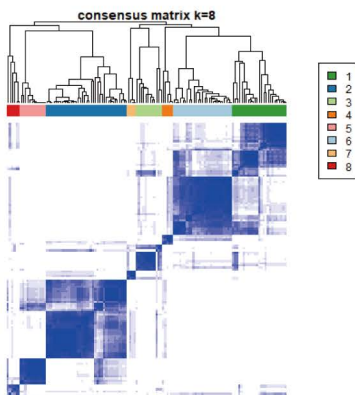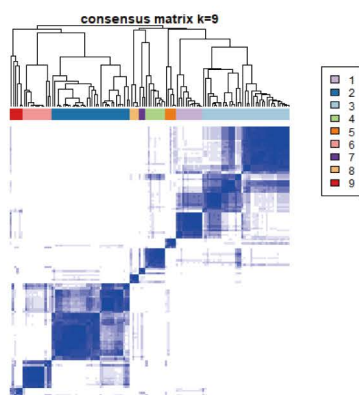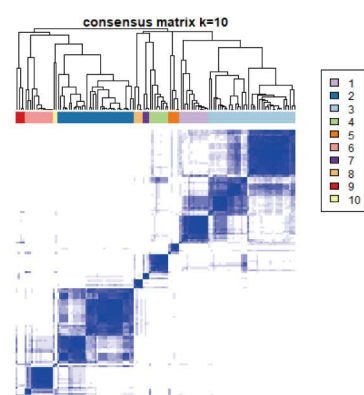

Supplement: Supplementary file 1 [file genes-13-01913-s001.zip › genes-1922944-Figure S1.pdf]

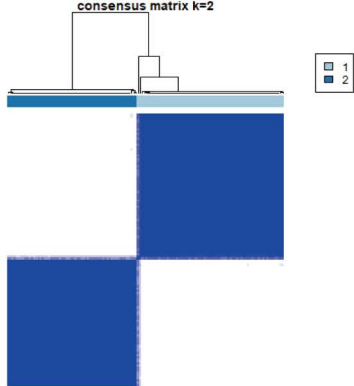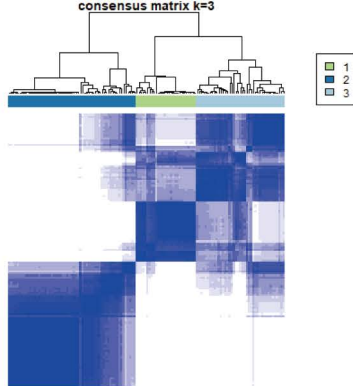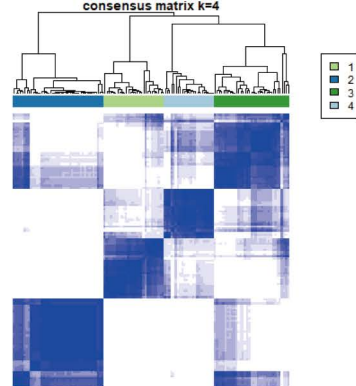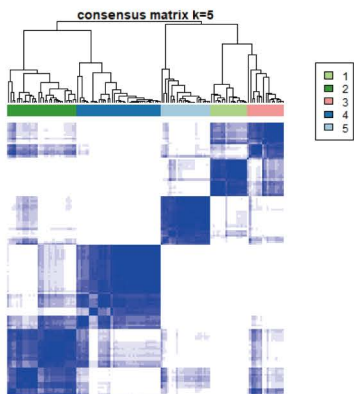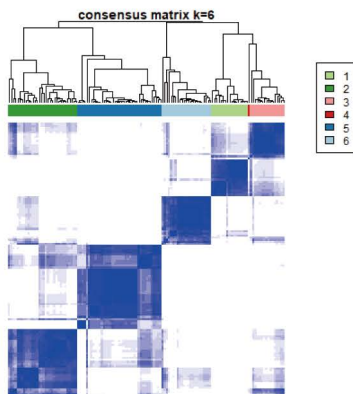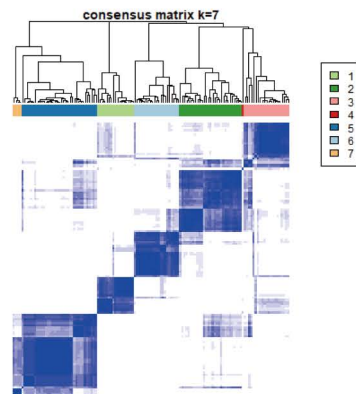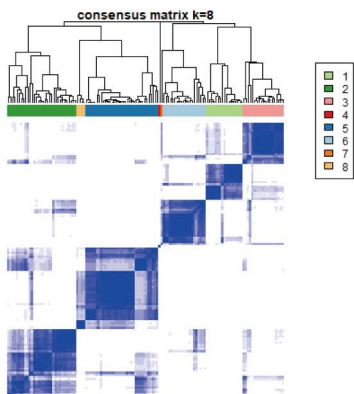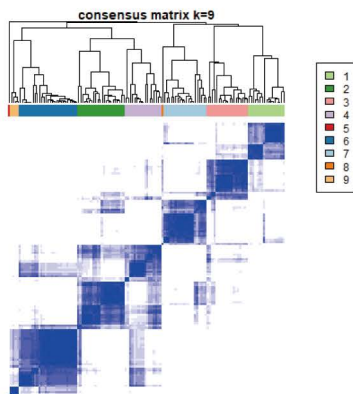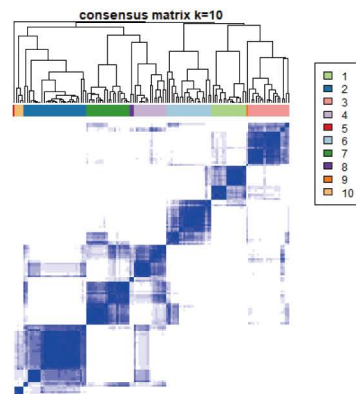

Supplement: Supplementary file 1 [file genes-13-01913-s001.zip › genes-1922944-Figure S2.pdf]
